# Quadrato Motor Training in Parkinson’s Disease: Resting-State fMRI Changes and Exploratory Whole-Brain Radiomics

**DOI:** 10.3390/bioengineering13050486

**Published:** 2026-04-22

**Authors:** Carlo Cosimo Quattrocchi, Claudia Piervincenzi, Raffaella Di Giacopo, Donatella Ottaviani, Maria Chiara Malaguti, Chiara Longo, Francesca Cattoi, Nikolaos Petsas, Loredana Verdone, Micaela Caserta, Sabrina Venditti, Bruno Giometto, Rossana Franciosi, Federica Vaccarino, Marco Parillo, Tal Dotan Ben-Soussan

**Affiliations:** 1 Centre for Medical Sciences-CISMed, University of Trento, 38122 Trento, Italy; carlo.quattrocchi@unitn.it (C.C.Q.); bruno.giometto@asuit.tn.it (B.G.); 2 Department of Radiological Sciences and Medical Imaging, Azienda Sanitaria Universitaria Integrata del Trentino (ASUIT) Provincia Autonoma Di Trento, 38123 Trento, Italy; francesca.cattoi@asuit.tn.it (F.C.); rossana.franciosi@asuit.tn.it (R.F.); 3 Department of Human Neurosciences, Sapienza University of Rome, 00185 Rome, Italy; claudia.piervincenzi@uniroma1.it; 4 Neurology Unit of Rovereto, Azienda Sanitaria Universitaria Integrata del Trentino (ASUIT) Provincia Autonoma Di Trento, 38123 Trento, Italy; raffaella.digiacopo@asuit.tn.it (R.D.G.); donatella.ottaviani@asuit.tn.it (D.O.); mariachiara.malaguti@asuit.tn.it (M.C.M.); chiara.longo@asuit.tn.it (C.L.); 5 School of Medical Statistics and Biometry, Department of Public Health and Infectious Diseases, Sapienza University of Rome, 00185 Rome, Italy; 6 Institute of Molecular Biology and Pathology, CNR, 00185 Rome, Italy; loredana.verdone@cnr.it (L.V.); mcaserta59@gmail.com (M.C.); 7 Department of Biology and Biotechnology “Charles Darwin”, Sapienza University of Rome, 00185 Rome, Italy; sabrina.venditti@uniroma1.it; 8 Research Institute of Neuroscience, Education and Didactics (RINED), Patrizio Paoletti Foundation, 06081 Assisi, Italy; research@fondazionepatriziopaoletti.org; 9 Ipazia LTD Higher Education Institution, Triq San Leonardu, XJR 2306 Xgħajra, Malta

**Keywords:** radiology, magnetic resonance imaging, resting-state fMRI, artificial intelligence, radiomics, neurology, Parkinson’s disease, brain, psychology, quadrato motor training

## Abstract

Parkinson’s disease (PD) may benefit from non-pharmacological motor–cognitive rehabilitation, but sensitive neuroimaging markers of training-related brain changes remain limited. This study investigated whether 4 weeks of daily Quadrato Motor Training (QMT) modulate resting-state functional connectivity (FC) in PD and secondarily explored whether whole-brain radiomic features derived from T1-weighted and fractional anisotropy (FA) images could detect pre–post differences over this short intervention interval. Fifty patients with idiopathic PD were randomized to QMT or a SHAM repetitive stepping condition, and 48 completed the protocol (25 SHAM, 23 QMT). MRI was acquired at baseline and after 4 weeks and included resting-state fMRI, 3D T1-weighted imaging, and diffusion-derived FA maps. Resting-state fMRI was analyzed using independent component analysis and dual regression, whereas an IBSI-compliant radiomics workflow and machine-learning models were used for exploratory scan-level classification. Compared with baseline, the SHAM group showed reduced synchronization across several resting-state networks, whereas the QMT group showed increased synchronization in the right sensorimotor and frontoparietal networks and no significant reductions. Between-group analyses showed lower delta-FC in SHAM than QMT in the cerebellar and sensorimotor networks. In contrast, radiomics showed limited discrimination between pre- and post-QMT scans; the best model achieved a ROC-AUC of 0.65 with near-chance accuracy, and no selected predictor remained significant after multiple-comparison correction. These findings suggest that QMT may support short-term functional network stability or task-relevant reorganization in PD relative to the SHAM condition, whereas whole-brain structural radiomics appears less sensitive for detecting early training-related effects in this setting.

## 1. Introduction

Parkinson’s disease (PD) is the second most common neurodegenerative disorder after Alzheimer’s disease, marked by motor symptoms such as resting tremor, rigidity, bradykinesia and postural instability, along with a range of non-motor manifestations, including cognitive impairment, depression, and autonomic dysfunction [1,2,3]. PD pathogenesis is multifactorial and involves abnormal α-synuclein aggregation, mitochondrial dysfunction, oxidative stress, impaired proteostasis, and neuroinflammatory processes, which together contribute to progressive dysfunction of dopaminergic and extranigral networks. In addition, emerging evidence suggests that metabolic factors, including glycemic variability and hypoglycemic episodes, may also interact with parkinsonian syndromes and deserve consideration within a broader pathophysiological framework [1,4]. While PD management continues to rely primarily on pharmacological treatments, there is a growing body of evidence supporting non-pharmacological approaches, particularly motor and cognitive training. These strategies are increasingly recognized for their neuroprotective and rehabilitative potential, likely mediated by modulation of sensorimotor and cerebellar pathways [5,6,7,8,9,10]. In PD, reduced basal-ganglia dopaminergic function diminishes movement automaticity and increases reliance on cognitive control and goal-based motor learning; accordingly, structured training and exercise have been proposed to promote experience-dependent neuroplasticity within motor and cognitive circuitry in PD [11].

Quadrato Motor Training (QMT), developed by Patrizio Paoletti, is a sensorimotor training protocol designed to enhance coordination, attentional focus, and emotional well-being by integrating physical movement with cognitive engagement [12]. During QMT, participants move within a designated square space in response to specific verbal commands, thereby requiring continuous attentional monitoring, response selection, inhibition, and spatial updating [12]. Because it combines externally guided motor sequencing with ongoing cognitive control, QMT differs from conventional repetitive motor exercise and may be particularly relevant in PD, where movement execution increasingly depends on cognitive resources. QMT combines externally guided motor sequencing with continuous cognitive monitoring and response inhibition, engaging neuroplastic changes in areas and networks implicated in both motor control and executive function [13,14]. More broadly, QMT plausibly engages perception–action coupling and sensorimotor transformation processes by requiring rapid mapping between auditory cues and coordinated motor output. In line with this framework, previous neuroimaging research has implicated premotor, cerebellar, and auditory–motor transformation networks in cue-driven sensorimotor processing [15]. Previous work in healthy populations suggests that QMT can improve executive–cognitive performance, including greater cognitive flexibility and faster reaction times [13]. QMT has also been associated with markers of neuroplasticity, such as higher circulating proNGF and proBDNF levels [16,17], as well as structural brain adaptations including increased cerebellar volume and indices consistent with improved white-matter organization [18,19,20]. These structural effects appear to be regionally specific rather than diffuse. In addition, QMT has been related to functional reconfiguration of brain networks and to changes in neural oscillatory activity, particularly in the alpha band, which is implicated in sensorimotor integration [8]. For example, increased electroencephalographic alpha coherence over frontal and parietal regions has been associated with better cognitive and emotional regulation [8,21], while magnetoencephalography studies suggest that approximately one month of daily QMT can enhance cerebellar alpha activity and interhemispheric alpha coherence alongside improvements in cognitive and motor performance [22]. Despite these findings, the neural effects of QMT have not yet been investigated in PD.

Neuroimaging offers a valuable framework for assessing training-related brain changes in PD, although reliable biomarkers remain limited, especially when intervention effects are expected to be subtle and distributed across large-scale networks. A recent systematic review identified only a limited number of magnetic resonance imaging (MRI) studies in this field and highlighted substantial heterogeneity in imaging targets, intervention protocols, and analytic methods, ultimately calling for more standardized and quantitatively robust strategies to characterize rehabilitation-related brain effects in PD [9]. In parallel, controlled PD intervention studies using advanced diffusion MRI analyses suggest that training-related effects may be measurable over relatively short timescales (e.g., weeks), but are often subtle and spatially circumscribed rather than global [23,24]. This temporal and spatial profile suggests that short-term intervention effects may be more readily captured by functional measures than by global structural descriptors. While electroencephalography provides high temporal resolution and is well suited to the study of oscillatory dynamics, functional MRI (fMRI) offers superior spatial resolution, making it particularly suitable for investigating the distributed neural systems underlying motor and cognitive dysfunction in PD and their potential modulation by QMT. In this context, resting-state fMRI (rs-fMRI) is especially relevant because it enables the investigation of spontaneous brain activity and large-scale functional network organization without requiring task performance. This is particularly advantageous in PD, where motor and cognitive deficits may affect task execution and introduce performance-related confounds [25,26,27,28]. Resting-state functional connectivity (FC) alterations in PD have been described in several networks, including the default mode, frontoparietal, and sensorimotor networks, and have been linked to both motor and cognitive impairment [29,30]. Rs-fMRI may therefore provide a sensitive means of detecting training-induced neuroplasticity by capturing changes in functional interactions across the brain following an intervention such as QMT.

Alongside FC analysis, quantitative image-based approaches may offer complementary information on subtle training-related brain changes. Radiomics, defined as the high-throughput extraction of quantitative features from medical images [31,32], enables the derivation of high-dimensional descriptors of tissue intensity, texture, and morphology that may reveal patterns not appreciable on conventional visual inspection. For example, Oliveira et al. applied gray-level co-occurrence matrix texture features extracted from structural MRI and evaluated multiple classical machine-learning algorithms for Alzheimer’s disease diagnosis, illustrating the continued relevance of texture-based radiomics with established machine learning baselines in neurodegenerative imaging contexts [33]. Because radiomic analysis is highly sensitive to image acquisition, preprocessing, discretization, and feature definition, reproducibility remains a major concern. The Image Biomarker Standardization Initiative (IBSI) addressed this issue by providing consensus feature definitions and standardized processing recommendations for quantitative imaging biomarkers [34]. Applying an IBSI-aligned radiomics workflow to longitudinal MRI data in PD may therefore provide a transparent and replicable benchmark for future rehabilitation neuroimaging studies. However, over relatively short intervention intervals, structural training-related effects may be subtle, regionally specific, and not necessarily detectable through whole-brain radiomic summaries. Accordingly, in the present study, radiomics was conceived as an exploratory complementary approach rather than as a modality expected a priori to show robust short-term sensitivity comparable to rs-fMRI.

The aim of the present study was to investigate changes in resting-state FC following a four-week daily QMT intervention in patients with PD. We hypothesized that QMT would modulate FC within key motor and cognitive networks and that this easy-to-implement motor–cognitive training might help mitigate network-level alterations associated with PD, thereby providing further insight into its potential contribution to rehabilitation. As a secondary exploratory objective, we also examined whether whole-brain radiomic features extracted from 3D T1-weighted images and fractional anisotropy (FA) maps showed measurable differences between scans acquired before and after the four-week QMT intervention, while recognizing that global structural radiomic descriptors may have limited sensitivity to short-term training-related effects over this time scale.

## 2. Materials and Methods

### 2.1. Study Design and Patient Selection

This study was conducted in accordance with the principles of the Declaration of Helsinki, and written informed consent was obtained from all participants. The protocol received approval from the Ethics Committee of the Fondazione Policlinico Universitario Campus Bio-Medico (PAR 44.22; clinical trial registry number 2022.086).

We pre-screened 65 patients with idiopathic PD and enrolled a final sample of 50 patients [35], who were randomized using a 1:1 allocation ratio (www.random.org) into two groups. The QMT group included 25 patients who performed QMT daily for 4 consecutive weeks, whereas the control group included 25 patients who performed a simple step in any direction after each audio instruction (SHAM). At the time of informed consent, each participant received a random code corresponding to the generated sequence in the presence of the principal investigator. Codes ranged from C01 to C25 for the control group and from Q01 to Q25 for the QMT group. Patients and caregivers were blinded to intervention allocation.

The longitudinal protocol consisted of two time points: baseline, corresponding to the day of recruitment (T0), and after 4 weeks of daily training (control or QMT) (T1). A 4-week interval has been used in previous longitudinal studies focusing on functional training-related changes in PD [36,37] and has also proved sufficient to induce neuroplastic changes in healthy subjects performing QMT [12]. We therefore considered this interval appropriate for detecting early functional network modulation, while recognizing that it might be less sensitive for capturing diffuse structural changes.

Patient screening and enrollment were conducted from January 2023 to August 2023 at the Neurology Unit of the Azienda sanitaria universitaria integrata del Trentino (Rovereto–Trento, Italy). Eligibility criteria were: (i) age ≥ 45 years; (ii) Hoehn & Yahr stage ≤ 3.0; (iii) ability to ambulate independently for at least 10 m; and (iv) a stable, optimized antiparkinsonian medication regimen maintained for at least 4 weeks before study entry. Clinical assessments were performed both before and after the intervention while patients were in the ON-medication state. Patients were excluded in the presence of: (i) dementia, defined as a corrected Mini-Mental State Examination score <24; (ii) current or previous peripheral sensory neuropathy; (iii) peripheral neurological, musculoskeletal, or orthopedic disorders potentially affecting gait and/or balance; (iv) lower-limb injury within the preceding 6 months; (v) prior neurosurgical or orthopedic surgery; (vi) history of epilepsy; (vii) use of medications that could influence cognitive and/or motor performance; (viii) history of depression or other psychiatric conditions; (ix) any absolute contraindication to MRI; (x) morphological MRI evidence of small-vessel ischemic disease or imaging findings suggestive of secondary parkinsonism; or (xi) implantation of a deep brain stimulation device.

The interval between 24 September 2023 and 25 October 2023 refers to the operational study window during which the enrolled participants underwent baseline assessment/MRI (pre-QMT), completed the 4-week intervention, and underwent follow-up assessment/MRI (post-QMT).

### 2.2. Interventions

**Figure 1:**
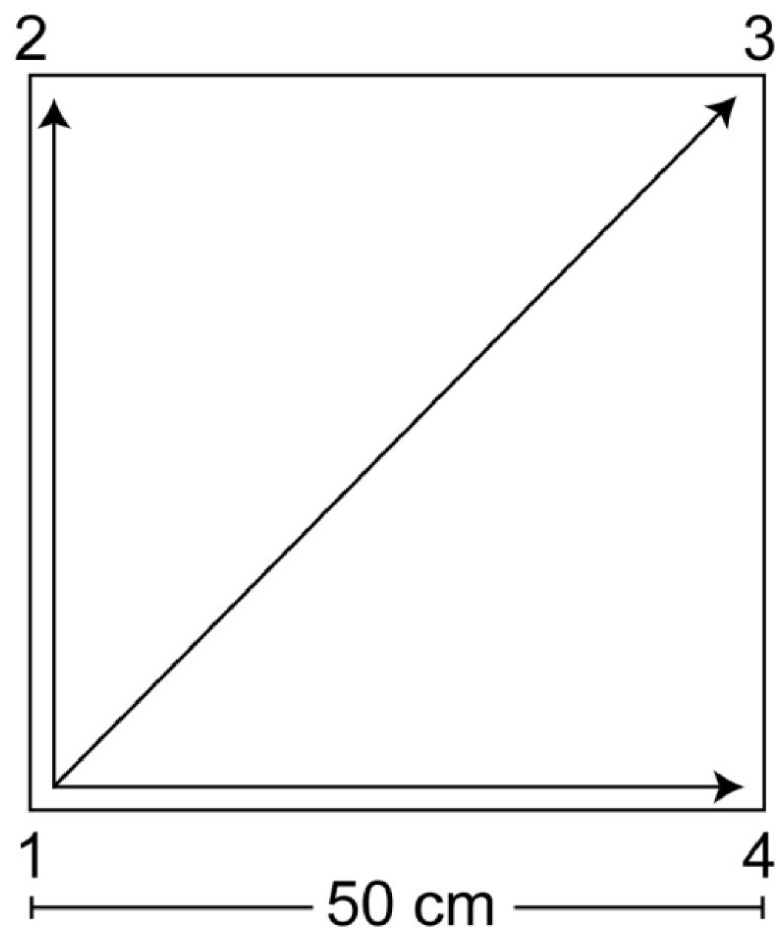
Visual representation of the Quadrato Motor Training (QMT). Participants stood at one corner of a 0.5 m × 0.5 m square in a quiet room and moved to different corners of the square in response to verbal instructions from an audio recording. The recording indicated the next corner to move to (for example, the command “one four” meant moving from corner 1 to corner 4). Participants were instructed to keep their eyes focused straight ahead, their hands loose at the side of the body, and initiate each movement with the leg closest to the center of the square. This experimental protocol involved daily training sessions, consisting of a sequence of 69 commands lasting 7 min.

QMT, originally created by Patrizio Paoletti, is performed while standing at a corner of a 0.5 m × 0.5 m square. Guided by an audio track, the participant executes single-step displacements from one corner to another according to the spoken cues, which indicate the next target position (Figure 1).

At each corner, three step choices are available, resulting in 12 possible displacements overall (3 options × 4 corners), including forward, backward, lateral (left/right), and diagonal moves (2 forward, 2 backward, 2 left, 2 right, and 4 diagonal steps). When the audio cue repeats the same number (e.g., “1–1”), participants must suppress the tendency to move and remain in place until the subsequent instruction. This stop/no-step component, requiring both cognitive and motor inhibition and rapid selection of the appropriate response based on the processed verbal cue, represents a core element of QMT. Participants were asked to (i) look straight ahead with arms relaxed alongside the body, (ii) proceed immediately to the next cue, and (iii) avoid pausing even if an error occurred. Each daily session comprised a 7-min sequence with 69 cues delivered at an average rate of ~0.5 Hz, corresponding to a slow walking cadence. Steps were initiated with the foot closest to the center of the square. All participants performed the same standardized audio protocol, ensuring identical cue sequence and pacing.

The setting and audio recording used for the SHAM exercise were the same as those used for the QMT group; however, the patients allocated in the control group were instructed to perform a step forward or backward at every command and without the use of a square.

### 2.3. Demographics and Clinical Data Collection

At recruitment, experienced neurologists (RDG, MCM, DO) assessed inclusion criteria and verified the willingness of the patient to participate. At T0 demographics and clinical data were collected. The database included the following variables: age, sex, marital status, weight, height, level of education, comorbidities, lifestyle such as diet and sports activities, handedness, score obtained at a self-efficacy questionnaire. Clinical data included: disease duration, Hoehn and Yahr scale, levodopa equivalent daily doses (LEDD), Movement Disorder Society sponsored Unified Parkinson’s Disease Rating Scale (MDS-UPDRS: Part I, II, III and IV), Time Up and Go test, number of falls in the month prior to this study. Clinical data were collected at T1 as well to monitor any change in patient status, although unexpected based on the natural history of the disease.

Based on the short time span of this study, the neuropsychological tests were performed only once investigating multiple domains: among those, global cognition using the Montreal Cognitive Assessment (MoCA) [38] was assessed.

### 2.4. Patient Notebook

We provided a notebook for every participant to record data linked to the daily practice. Particularly, patients were asked to record daily about the completion and duration of the training, the concomitant use of additional drugs during the training period, the level of satisfaction after the training using a 3-point Likert scale (low, moderate, high), the number of hours slept the night before the training.

### 2.5. MRI Protocol

MRI data were acquired at T0 and T1 on a 1.5 T Magnetom Aera system (Siemens, Erlangen, Germany) using a 12-channel head matrix coil.

Whole-brain rs-fMRI (230 volumes, 9 min 51 s) was acquired with echo-planar imaging with prospective acquisition correction, 76 contiguous axial slices parallel to the anterior–posterior commissure plane with interleaved multislice T2* echo-planar imaging. Field mapping was obtained using a double echo gradient echo (GRE) T2*-weighted sequence.

We also analyzed a high-resolution T1-weighted 3D Magnetization-Prepared Rapid Gradient-Echo (MPRAGE) sequence to characterize macroscopic brain anatomy and tissue contrast, providing a robust substrate for whole-brain radiomic feature extraction. In parallel, we used FA maps derived from diffusion tensor imaging to capture microstructural properties of white matter organization, which may be sensitive to training-related changes not detectable on conventional structural imaging (Appendix A).

Before the scan, patients were instructed to relax, keep their eyes closed, and stay as still as possible without falling asleep.

An expert in neuroradiology (CCQ) examined all MRIs, to exclude the presence of concomitant brain lesions and focal white matter hyperintensities according to the Fazekas scale [39].

### 2.6. rs-fMRI Data Analysis

Anatomical and functional preprocessing were performed using fMRIPrep 20.2.7 [40] (RRID:SCR_016216), which is based on Nipype 1.7.0 [41,42] (RRID:SCR_002502) and FSL v6.0.4 (https://fsl.fmrib.ox.ac.uk/fsl/docs/#/, latest access on 7 March 2025).

Each of the T1-weighted images was preprocessed with the following pipeline: First, the T1-weighted image was corrected for intensity non-uniformity (INU) with N4BiasFieldCorrection [43], distributed with ANTs 2.3.3 [44] (RRID:SCR_004757), and used as T1-weighted-reference throughout the workflow. The T1-weighted-reference was then skull-stripped with a Nipype implementation of the antsBrainExtraction.sh workflow (from ANTs), using OASIS30ANTs as the target template. Brain tissue segmentation of cerebrospinal fluid (CSF), white-matter (WM) and gray-matter (GM) was performed on the brain-extracted T1-weighted using fast [45] (FSL 5.0.9, RRID:SCR_002823). Brain surfaces were reconstructed using recon-all [46] (FreeSurfer 6.0.1, RRID:SCR_001847), and the brain mask estimated previously was refined with a custom variation of the method to reconcile both ANTs-derived and FreeSurfer-derived segmentation of the cortical gray-matter of Mindboggle [47] (RRID:SCR_002438). Volume-based spatial normalization to two standard spaces (MNI152NLin2009cAsym, MNI152NLin6Asym) was performed through nonlinear registration with antsRegistration (ANTs 2.3.3), using brain-extracted versions of both T1-weighted reference and the T1-weighted template. The following templates were selected for spatial normalization: ICBM 152 Nonlinear Asymmetrical template version 2009c [48] (RRID:SCR_008796; TemplateFlow ID: MNI152NLin2009cAsym), FSL’s MNI ICBM 152 non-linear 6th Generation Asymmetric Average Brain Stereotaxic Registration Model [49] (RRID:SCR_002823; TemplateFlow ID: MNI152NLin6Asym].

For each subject, the following preprocessing was performed: First, a reference volume and its skull-stripped version were generated using a custom methodology of fMRIPrep. A B0-nonuniformity map (or fieldmap) was estimated based on a phase-difference map calculated with a dual-echo GRE sequence, processed with a custom workflow of SDCFlows inspired by the epidewarp.fsl script and further improvements in HCP Pipelines [50]. The fieldmap was then co-registered to the target echo-planar imaging (EPI) reference run and converted to a displacement field map (amenable to registration tools such as ANTs) with FSL’s fugue and other SDCflows tools. Based on the estimated susceptibility distortion, a corrected EPI reference was calculated for a more accurate co-registration with the anatomical reference. The blood-oxygen-level-dependent (BOLD) reference was then co-registered to the T1-weighted reference using bbregister (FreeSurfer) which implements boundary-based registration [51]. Co-registration was configured with six degrees of freedom. Head-motion parameters with respect to the BOLD reference (transformation matrices, and six corresponding rotation and translation parameters) are estimated before any spatiotemporal filtering using mcflirt [52] (FSL 5.0.9). BOLD runs were slice-time corrected using 3dTshift from AFNI 20,160,207 [53] (RRID:SCR_005927). The BOLD time-series (including slice-timing correction when applied) were resampled onto their original, native space by applying a single, composite transform to correct for head-motion and susceptibility distortions. These resampled BOLD time-series will be referred to as preprocessed BOLD in original space, or just preprocessed BOLD. The BOLD time-series were resampled into standard space, generating a preprocessed BOLD run in MNI152NLin2009cAsym space. First, a reference volume and its skull-stripped version were generated using a custom methodology of fMRIPrep. Automatic removal of motion artifacts using independent component analysis (ICA-AROMA) [54] was performed on the preprocessed BOLD on MNI space time-series after removal of non-steady state volumes and spatial smoothing with an isotropic, Gaussian kernel of 6 mm FWHM (full-width half-maximum). Corresponding “non-aggresively” denoised runs were produced after such smoothing. Additionally, the “aggressive” noise-regressors were collected and placed in the corresponding confounds file. Functional preprocessed data were finally subjected to WM and CSF signal regression and high-pass filtering (100-s cut-off) [54].

Independent component analysis (ICA) of preprocessed functional data was performed using FSL’s MELODIC tool (Multivariate Exploratory Linear Optimized Decomposition into Independent Components) [41,55]. For group-wise ICA, a single 4D data set was created by temporally concatenating preprocessed functional data. Group ICA was performed using a dimensionality of 20 components [55]. Resting-state networks (RSNs) of interest were identified via spatial correlation coefficients (fslcc tool) using RSNs generated by Smith et al. [56] and Yeo et al. [57] templates, and then verified by expert visual inspection (CP, 10 years of experience and CCQ, 20 years of experience).

The set of spatial maps from the group-average analysis was used to generate subject-specific versions of the spatial maps and associated time series using a dual regression technique [58,59]. For each subject, the group-average set of spatial maps was first regressed (as spatial regressors in a multiple regression) into the subject’s 4D space-time dataset, resulting in a set of subject-specific time series, one per group-level spatial map. These time series were then regressed (as temporal regressors in a multiple regression) into the same 4D dataset, resulting in a set of subject-specific spatial maps, one per group-level spatial map. Individual difference maps between T1 and T0 (ΔFC maps) were also obtained for each RSN.

### 2.7. Radiomic-Based Machine-Learning Modelling

In this subgroup analysis on the axial FA and T1-weighted MPRAGE images, the dataset comprised 40 samples: 20 pre-QMT and 20 post-QMT. This image set was used to train, validate, and test five machine-learning models in a binary classification framework (“pre-QMT” versus “post-QMT”). The supervised learning task was intentionally formulated as an exploratory scan-level classification problem aimed at discriminating examinations acquired at the two timepoints, rather than as a subject-level longitudinal prediction model. Although each subject contributed two scans (pre and post), we did not compute subject-specific change features (post–pre delta features) nor implement paired longitudinal modelling. Instead, scans acquired at the two timepoints were treated as two groups for model training and evaluation. This design does not account for within-subject dependence and therefore does not support subject-level longitudinal generalizability; accordingly, model performance should be interpreted strictly as an exploratory within-cohort feasibility estimate. No formal a priori power calculation was performed specifically for the radiomics workflow, as this component of this study was conceived as an exploratory feasibility analysis rather than a definitive predictive modelling study.

Radiomic methodology was applied to the collected images according to the IBSI guidelines (https://arxiv.org/abs/1612.07003, accessed on 7 December 2025). For this purpose, the Trace4Research™ platform (version n. 2.1.02, DeepTrace Technologies S.R.L.) was used to obtain an IBSI-compliant radiomic workflow. The complete workflow included: (1) segmentation of the volume of interest (VOI) from each image, (2) preprocessing of image intensities within the segmented VOI, (3) computation of radiomic features, (4) selection of informative and non-redundant radiomic features, (5) outlier analysis, (6) training, validation, and testing of different supervised machine-learning classifiers for the binary classification task (“pre-QMT” versus “post-QMT”), and (7) model calibration analysis.

**Figure 2:**
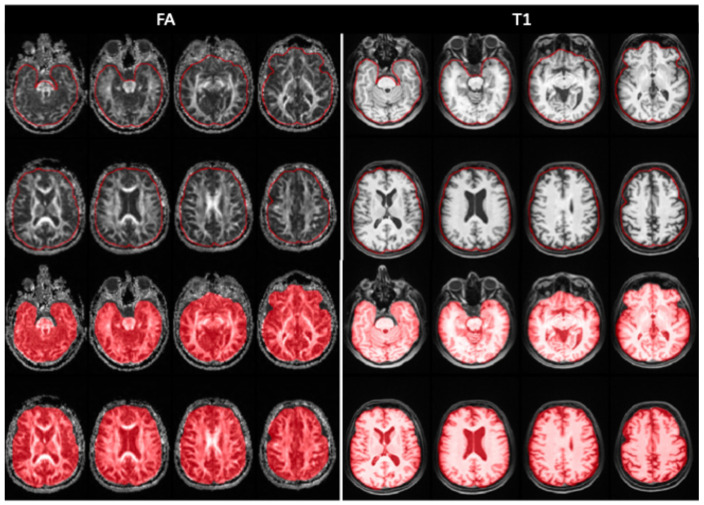
Representative examples of whole-brain segmentation on MRI fractional anisotropy (FA) images (**left**) and T1-weighted MPRAGE images (**right**). For each modality, both the segmentation contour (red outline) highlighting the boundaries of the segmented volume and the segmented mask (red overlay) for localization within the whole brain are shown.

More specifically:

(1) The segmentation of the VOI was performed manually, slice by slice, using the Trace4Research segmentation tool. Whole-brain segmentation was performed by a primary rater (radiologist with 5 years of experience in neuroimaging), who delineated all VOIs across the entire internal dataset (Figure 2). A second radiologist (8 years of experience) independently reviewed all segmentations for anatomical plausibility and adherence to the segmentation protocol. Any uncertainties or disagreements were resolved by consensus. The VOI encompassed the entire brain parenchyma, including both cerebral hemispheres, brainstem, and cerebellum. During segmentation, gross extra-parenchymal cerebrospinal fluid spaces, particularly enlarged subarachnoid spaces, were excluded whenever clearly identifiable, to minimize inclusion of non-parenchymal signal.

(2) Preprocessing of image intensities within the segmented VOI included resampling to a fixed isotropic voxel spacing to ensure a consistent spatial grid across modalities for radiomic feature extraction. T1-weighted 3D MPRAGE images were acquired at 1 × 1 × 1 mm^3^ and were therefore retained at native resolution, whereas FA maps (native voxel size 2 × 2 × 2 mm^3^) were resampled to 1 × 1 × 1 mm^3^ using linear interpolation. FA maps were resampled to 1 mm isotropic voxels to standardize voxel geometry and improve comparability of spatial relationships for texture feature computation, consistent with common radiomics recommendations on voxel resampling [60,61,62]. VOI masks were resampled using nearest-neighbor interpolation to preserve label integrity. All preprocessing steps were applied identically to pre-QMT and post-QMT scans to minimize acquisition-related confounding.
(3) The Radiomics features computed from the segmented VOI belonged to different families: Morphology, Intensity-based Statistics, Intensity Histogram, Gray-Level Co-occurrence Matrix (GLCM), Gray-Level Run Length Matrix (GLRLM), Gray-Level Size Zone Matrix (GLSZM), Neighborhood Gray Tone Difference Matrix (NGTDM), Neighboring Gray Level Dependence Matrix (NGLDM). Such features were extracted from the original image and its filtered versions (square, squareroot, exponential, and logarithm filters), except for the features of the family Morphology that were extracted just from the original image. Their definition, computation, and nomenclature are compliant with the IBSI guidelines. Steps from (2) to (3) were performed using the Trace4Research Radiomics tool. It must be noted that, for both sequences, Intensity Histogram features were computed after an intensity discretization of the VOI, using a fixed number of 64 bins. Texture features (GLCM, GLRLM, GLSZM, NGTDM, NGLDM) were computed after an intensity discretization of the VOI, using a fixed number of 64 bins, commonly used in radiomics studies. Discretization is required for most texture matrices and the fixed number of 64 bins represents a pragmatic trade-off between sensitivity to intensity variation and robustness to noise [60,61,62]. Radiomic features were reported by Trace4Research according to IBSI standards.
(4) Features with low coefficient of variation (threshold = 0.1) and low mutual-information with the class label (threshold = 0.3) were removed. To address the issue of intercorrelated features, a mutual-information analysis was conducted, using a genetic algorithm to optimize a custom fitness function. This function operates on a selected set of candidate features and takes into account two factors: the mean intercorrelation, expressed as symmetric uncertainty among the discretized features (12 bins), and the mean correlation of each feature with the class label, also expressed as symmetric uncertainty. Therefore, the resulting set of features is chosen to maximize useful information and minimize redundancy. The selected radiomic features (informative and non-redundant) were reported by Trace4Research according to IBSI standards.
(5) Outlier analysis was conducted on the selected features, using Mahalanobis distance to identify outliers, considering a threshold of 3 standard deviations from the mean.
(6) Five different machine-learning architectures were trained, validated across multiple hyperparameter configurations, and tested, for the binary classification task of interest (“post-QMT” versus “pre-QMT”), based on supervised learning, using the timepoint label as the ground-truth class. For each architecture, three ensembles were trained with a nested random 10-fold cross validation approach, therefore each model consisted of three ensembles of 100 classifiers. The first model consisted of Random Forest classifiers combined with Gini Index, Principal Components Analysis (PCA), and Fisher Discriminant Ratio (FDR) with mean-vote rule; the second model consisted of Support Vector Machine classifiers combined with PCA and FDR with mean-vote rule; the third model consisted of K-Nearest Neighbors classifiers combined with PCA and FDR with mean-vote rule; the fourth model consisted of Multi-Layer Perceptron classifiers combined with PCA and FDR with mean-vote rule; the fifth model consisted of Logistic Regression classifiers combined with PCA and FDR with mean-vote rule. We selected these models as well-established classical baselines commonly used in radiomics studies. They provide a pragmatic balance of interpretability and capacity for non-linear decision boundaries and are frequently adopted in radiomics because typical radiomics datasets are high-dimensional and comparatively small, making “classical” supervised learners appropriate and informative benchmarks [61,63,64]. Our goal was not to claim state-of-the-art performance, but to provide a transparent baseline comparison and an empirical feasibility benchmark under commonly used radiomics classifiers. For each model, a Grid Search was performed to select the best hyper-parameters, based on the validation set performance. To avoid information leakage across datasets, the following processing steps were performed after the cross-validation split: features normalization, PCA, model training and optimization. The performance of the five models was measured for each classifier in terms of Area Under the Receiver Operating Characteristic Curve (ROC-AUC), Accuracy, Sensitivity, Specificity, Positive Predictive Value (PPV), Negative Predictive Value (NPV), F1 score. For the computation of such performance metrics, the class “post-QMT” was considered as the Positive class. The results were then averaged, also computing 95% confidence intervals (CI) and *p*-values for one-sided Wilcoxon signed rank test, performed to assess statistical significance with respect to chance/random classification. The significance levels were set at 0.05 (*) and 0.005 (**). The model with the best performance, according to mean ROC-AUC, on the internal testing set was chosen as the best model for the binary task of interest (“post-QMT” versus “pre-QMT”).
(7) Model calibration analysis was conducted for each of the five different machine-learning architectures. Reliability diagrams were used to assess the alignment between predicted probabilities and observed outcomes, applying a fixed number of 10 equally spaced bins. Additionally, Expected Calibration Error (ECE) and Brier Score (BS) were calculated.

### 2.8. Statistical Analysis

Statistical analyses of demographic, clinical and neuropsychological parameters were performed using SPSS statistics software (version 22.0). Between-group differences at T0 were tested using Mann–Whitney U test and Fisher’s exact test for continuous and dichotomous variables, respectively (*p* < 0.05 for null hypothesis rejection).

For network-based FC, subject-specific spatial maps obtained from dual regression analysis were entered into group-level voxel-wise analyses. For each RSN, we investigated FC differences at T0, by comparing the 2 patient groups using a two-sample unpaired t test. To investigate FC changes after 4 weeks of training (Control or QMT) and to compare longitudinal changes between the 2 patient groups, a two-sample unpaired t-test was performed on ΔFC maps of the 2 patient groups. Age, sex, and total intracranial volume were used as covariate of no interest in all analyses. Voxel-wise statistical analyses were performed with permutation-based non-parametric statistics using FSL Randomize permutation-based program with 5000 permutations. Results were corrected using false discovery rate (FDR) correction [65] for multiple comparisons (*p* < 0.01). Anatomical localization of significant clusters was established according to the Harvard–Oxford Cortical Structural Atlas included in FSL (http://www.fmrib.ox.ac.uk/fsl/data/atlas descriptions.html, latest access on 7 March 2025).

For radiomic assessment, statistical analysis was conducted with embedded tools of the Trace4Research platform. To describe the distribution of each of the most relevant features in the “post-QMT” and “pre-QMT” classes, we calculated the median values with 95% CI and presented graphically for intuitive visualization and interpretation. A non-parametric univariate Wilcoxon rank-sum test (Mann-Whitney U test) was performed for each of the relevant radiomic predictors to verify its significance in discriminating “post-QMT” and “pre-QMT” classes. To account for multiple comparisons, the *p*-values were adjusted using the Bonferroni method. For all the *p*-values reported in this manuscript, significance levels were set at 0.05 (*) and 0.005 (**). Because the radiomics analysis was implemented at the scan level, these univariate comparisons should also be interpreted as descriptive between-timepoint comparisons within the study cohort rather than as paired subject-level longitudinal inference.

## 3. Results

### 3.1. Enrolled Patients

**Figure 3:**
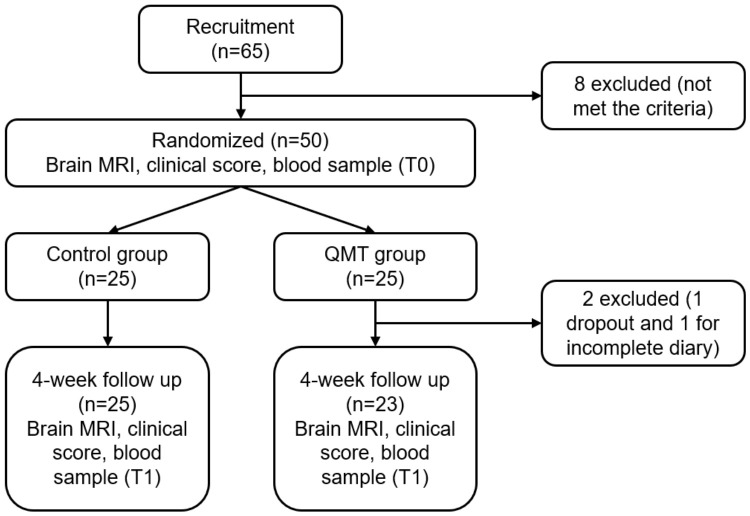
Flowchart of this study.

Twenty-five patients of the control group and 23 patients in the QMT group completed the protocol (Figure 3).

At T0, Control and QMT patient groups were similar in terms of socio-demographic and clinical features and performed similarly in all cognitive tests at the standard neuropsychological assessment. Demographic, clinical, and significant psychological features are shown in Table 1. At T1, no adverse events were reported during the QMT sessions. Clinical follow-up measures collected at T1 did not show statistically significant pre/post differences over the 4-week interval in either group (all *p* > 0.05).

### 3.2. Resting-State FC

**Figure 4:**
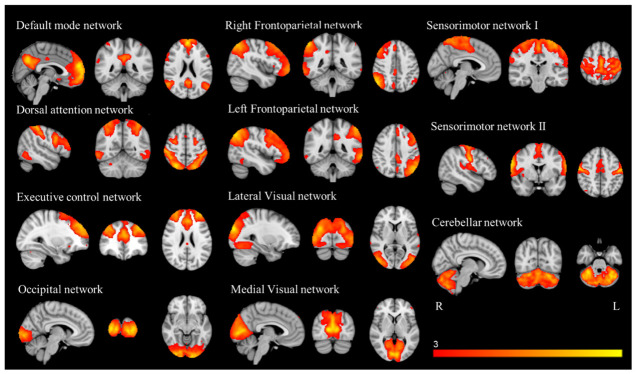
Resting-state networks (RSNs) identified and used for dual regression analysis. This figure shows sagittal, coronal, and axial slices for the RSNs detected, overlaid onto the MNI152 standard brain. RSNs are shown in FSL red-yellow color encoding using a 3 < z-score < 10 threshold window.

ICA yielded 20 independent components representing group-averaged networks of brain regions with temporally correlated BOLD fMRI signals. Of these, we identified 11 components that showed the highest spatial correlation coefficients with RSN templates: default mode (r = 0.59), left and right frontoparietal (r = 0.60 and r = 0.58, respectively), dorsal attention (r = 0.53), executive control (r = 0.45), lateral visual (r = 0.64), medial visual (r = 0.76), occipital (r = 0.71), cerebellar (r = 0.43) and two sensorimotor (r = 0.44 for sensorimotor I and r = 0.55 for sensorimotor II, respectively) networks (Figure 4).

At T0, no significant difference in intra-network synchronization was found in any RSN between Control and QMT groups.

**Figure 5:**
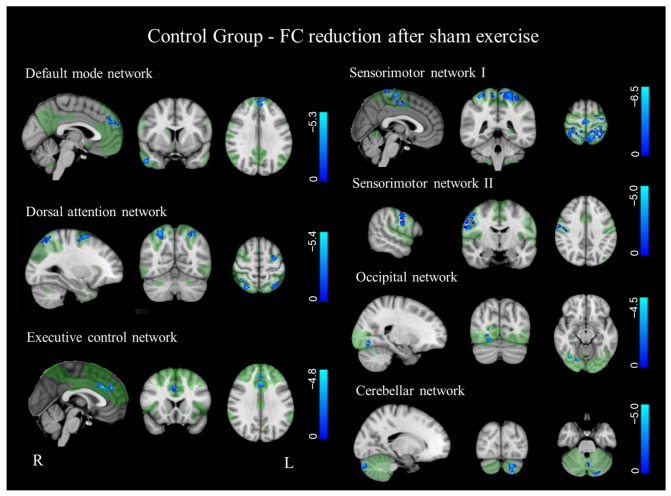
Significantly reduced FC in SHAM group (*p* < 0.01, FDR corrected). Results for each RSN are overlaid onto the corresponding network (green) in the MNI152 standard brain. Blue-light blue indicates areas of reduced FC in SHAM group. The color bars represent t values.

After 4 weeks of repetitive movement, the Control group showed significantly decreased synchronization (*p* < 0.01 FDR corrected) in regions of several RSNs, including default mode, dorsal attention, executive control, occipital, cerebellar and both sensorimotor networks (Figure 5, Table 2).

**Figure 6:**
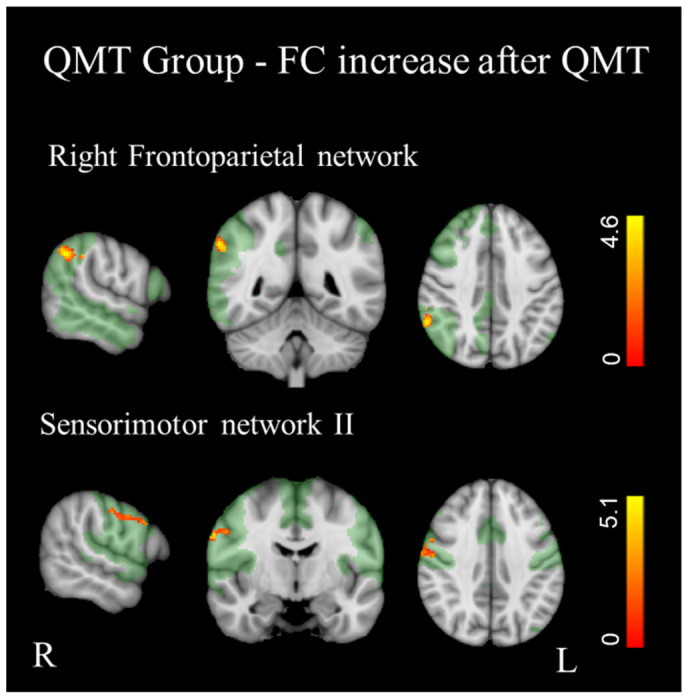
Significantly increased FC in QMT group (*p* < 0.01, FDR corrected). Results for each RSN are overlaid onto the corresponding network (green) in the MNI152 standard brain. Red-yellow indicates areas of increased FC in QMT group. The color bars represent t values.

Instead, the QMT group exhibited significantly increased synchronization (*p* < 0.01 FDR corrected) in the sensorimotor II network, particularly in the right pre- and post-central gyri, and in the right frontoparietal network, specifically in the right angular gyrus (Figure 6, Table 3).

In the QMT group, we did not find a significant reduction in activity synchronization in any of the RSN.

**Figure 7:**
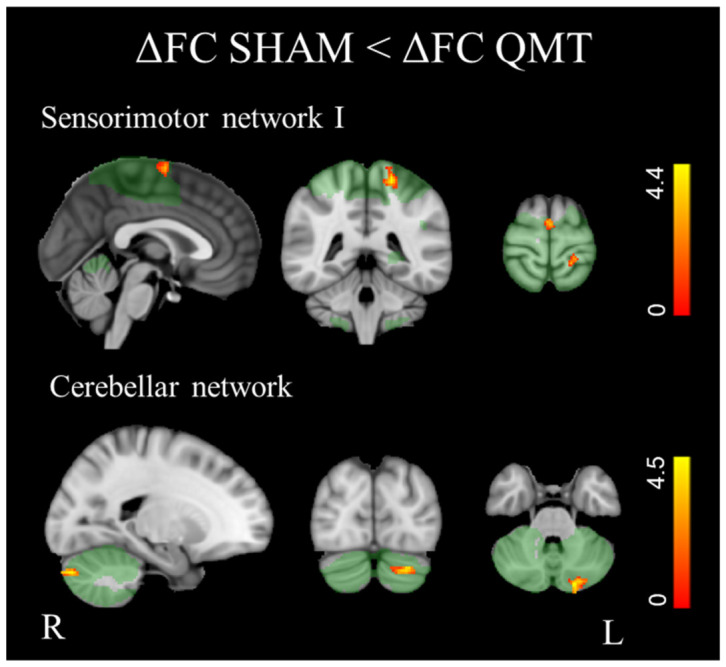
Significant differences between ΔFC maps in SHAM and QMT groups (*p* < 0.01, FDR corrected). Red-yellow areas indicate where FC was significantly lower in SHAM patients than in QMT. The color bars represent t values.

When the ΔFC maps of the two groups were compared, the Control group demonstrated a significantly lower (*p* < 0.01 FDR corrected) FC compared to the QMT group in the cerebellar network, particularly in the left Crus II, and in the sensorimotor I network, in the left post-central gyrus and in the supplementary motor cortex (Figure 7, Table 4).

### 3.3. Radiomic-Based Machine-Learning Models

**Figure 8:**
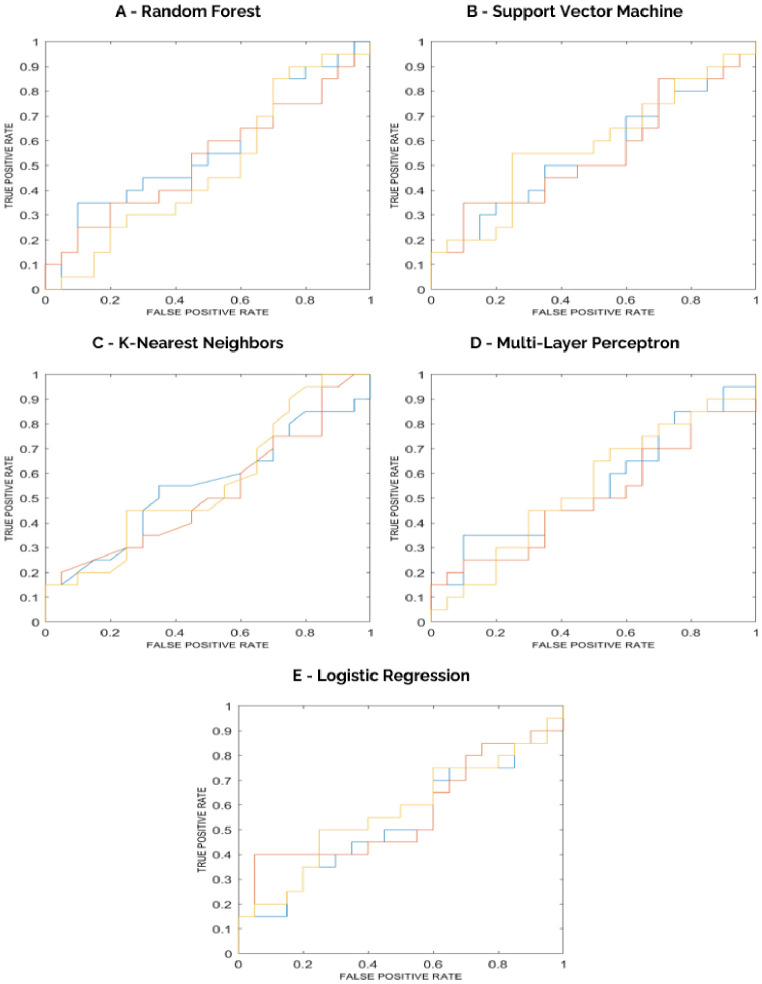
ROC curves for each ensemble of the models, from internal testing with aggregated predictions (**A**–**E**).

IBSI-compliant radiomic features were extracted from the whole-brain VOIs on axial FA and axial T1-weighted images, yielding a total of 1160 features (580 from FA and 580 from T1). After feature reduction, 6 predictors were retained. Outlier analysis on the final feature set identified 1 outlier in the “post-QMT” group and none in the “pre-QMT” group. The 6 selected features were used to train, validate, and test five supervised classifiers (Random Forest, Support Vector Machine, K-Nearest Neighbors, Multi-Layer Perceptron, Logistic Regression) using nested 10-fold cross-validation for the binary task “post-QMT” versus “pre-QMT”. Performance results are reported in Table 5, Table 6, Table 7, Table 8 and Table 9, while the corresponding ROC curves are shown in Figure 8.

Based on internal testing ROC-AUC, the Support Vector Machine model provided the best performance, with ROC-AUC 65% [54–76] and Accuracy 49% [42–56], along-side Sensitivity 52% [38–65] and Specificity 47% [32–61] (“post-QMT” as positive class).

Although the best-performing model yielded ROC-AUC 65%, threshold-dependent metrics (accuracy and F1 score) were near chance at the default decision threshold. This discrepancy reflects the threshold-free nature of AUC versus the sensitivity of accuracy/F1 to threshold choice in the presence of substantial overlap between classes; therefore, these results indicate limited separability of pre/post scans and should be interpreted as a pilot feasibility benchmark [61,66]. Importantly, because the modelling strategy was implemented at the scan level and did not explicitly model within-subject dependence, these results should not be interpreted as evidence that radiomic features can reliably classify longitudinal change at the individual-patient level.

**Figure 9:**
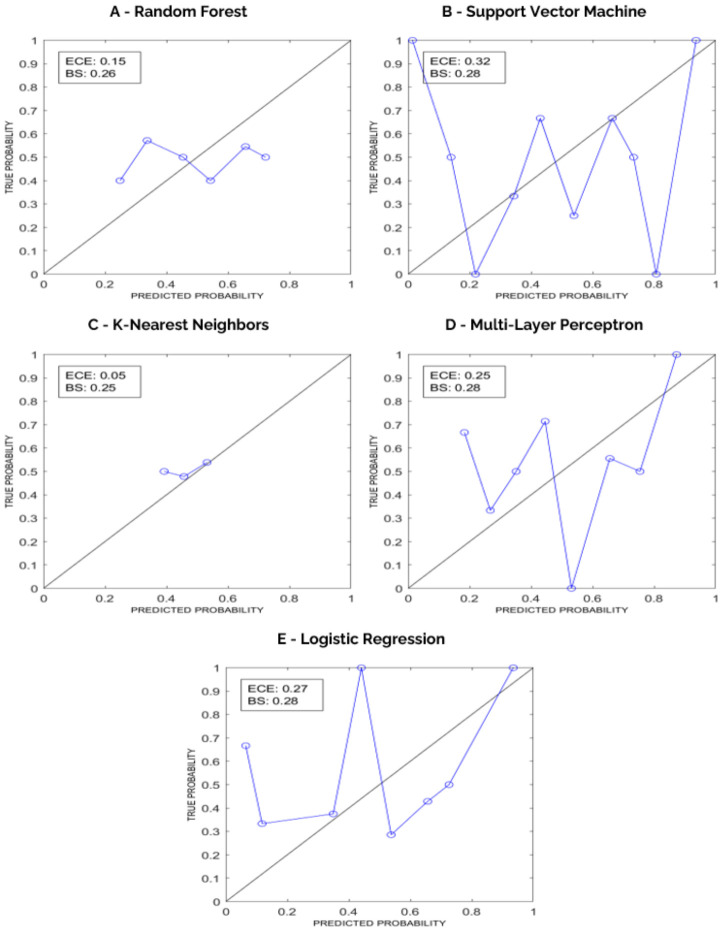
Reliability diagrams for each model, from internal testing with aggregated predictions. Predicted probability (consensus) is compared with true probability of being in the positive class. ECE and BS are reported (**A**–**E**).

Calibration results are presented in Figure 9, with reliability diagrams and the corresponding ECE and BS values.

**Figure 10:**
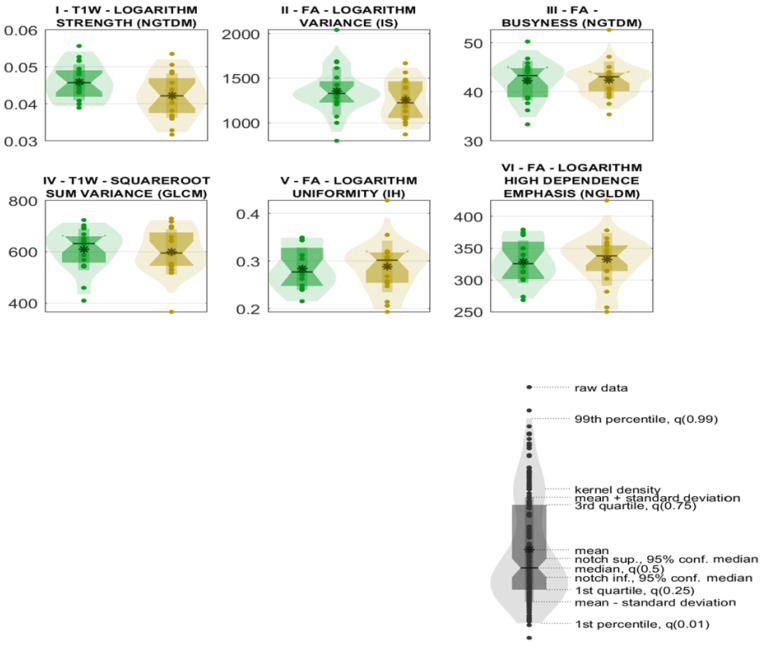
Distribution plots of the 6 selected predictors. Distribution plots of “post-QMT” and “pre-QMT” classes are reported in gold and green, respectively.

The six selected predictors are summarized in Table 10, including feature family, nomenclature, and group-wise medians with 95% confidence intervals; their distributions are shown in Figure 10.

After Bonferroni correction, none of the univariate comparisons reached statistical significance (corrected *p*-values ranging from 0.61 to 1.00).

## 4. Discussion

The present study investigated the effects of QMT on brain organization in patients with PD by combining rs-fMRI with an exploratory whole-brain radiomics approach applied to structural MRI-derived data. Overall, the findings suggest that QMT may support relative functional stability and selective reorganization of large-scale brain networks in PD when compared with the SHAM condition, whereas whole-brain radiomic signatures derived from T1-weighted MPRAGE images and FA maps appear to provide only limited sensitivity for detecting short-term training-related effects over the studied interval.

With regard to rs-fMRI, two main observations emerged. After 4 weeks of intervention, the control group exhibited a reduction in region-to-region BOLD synchronization across most of the investigated networks, whereas the QMT group showed increased synchronization in selected regions of the sensorimotor and frontoparietal networks and relatively stable synchronization in the remaining networks compared with controls. This pattern suggests that QMT may favor relative network stability and focal enhancement of functional connectivity compared with the SHAM condition in networks relevant to both motor control and higher-order cognitive processing in PD. Such an interpretation is biologically plausible given the specific characteristics of QMT, which combines externally guided movement, spatial navigation, response selection, sustained attention, and inhibition. Because PD is characterized not only by impaired movement automaticity but also by increased reliance on cognitive control during action, an intervention that systematically engages both motor and executive processes may be particularly suited to promote adaptive network-level plasticity.

At the physiological level, resting-state synchronization detected by rs-fMRI is generally interpreted as the macroscopic correlate of coordinated infraslow neural population activity, coupled to vascular responses through neurovascular mechanisms rather than as a direct readout of simultaneous neuronal firing per se [67,68]. A purely neurocentric interpretation is likely incomplete, however, because neurovascular coupling depends on coordinated signaling among neurons, astrocytes, pericytes, and vascular cells, and astrocytes are anatomically well positioned to relay synaptic activity to the vasculature; consistent with this view, experimental work has shown that astrocytic Ca^2+^ signals can be coupled to both positive and negative BOLD responses [69,70]. In PD, glial and neuroinflammatory mechanisms may further influence large-scale brain function, suggesting that non-neuronal contributions to network dynamics deserve consideration even though their direct role in human RSN synchronization remains incompletely defined [71]. Importantly, homologous resting-state networks have been identified in rodents and non-human primates, and cross-species fMRI work supports the presence of evolutionarily conserved organizational principles, providing a translational framework for interpreting our sensorimotor, frontoparietal, and cerebellar findings in PD [72,73].

These findings are broadly consistent with previous rs-FC studies showing altered connectivity in PD compared with healthy individuals, including changes involving the inferior parietal lobule and supramarginal gyrus [74,75]. More generally, different motor rehabilitation programs in PD have been associated with measurable rs-FC changes in the supplementary motor area (SMA) [6], within the sensorimotor network [42], and in regions belonging to the dorsal attention and cerebellar networks [76,77]. Likewise, mechanical peripheral stimulation has been reported to induce stimulus-specific increases in connectivity in brain regions involved in visuospatial and sensorimotor integration [11]. Together, these studies suggest that training-related plasticity in PD is at least partly task-dependent, and that interventions engaging distinct motor and cognitive demands may produce different network-level signatures. In addition, exercise programs incorporating cognitive engagement have consistently been associated with improvements in gait and balance in PD [78], while interventions such as treadmill training, Tai Chi, tango dancing, boxing, and forced cycling have also demonstrated benefits in this population [79,80,81,82,83,84,85]. Within this broader framework, the present results support the view that QMT belongs to a class of motor-cognitive interventions capable of modulating large-scale functional systems in PD.

A particularly relevant aspect of the present study is the divergent trajectory observed between the two intervention arms. Although no significant between-group differences were detected at baseline, the control group showed a post-intervention reduction in synchronization in networks previously described as vulnerable in PD [27]. However, given the short 4-week interval, these reductions should not be interpreted as reflecting true disease progression. An alternative and, in our view, more plausible explanation is that the SHAM condition induced a state of relative neural disengagement or habituation. Because the control exercise involved repetitive stepping with minimal cognitive demand, it may have provided insufficient stimulation to sustain the engagement of higher-order motor-cognitive networks. In this framework, the apparent advantage of QMT may reflect both active task-related network recruitment in the QMT arm and reduced network engagement in the SHAM arm. This interpretation remains cautious and inferential; however, it is also consistent with the qualitative reports of fatigue and boredom recorded in patient notebooks in the control group, which may have contributed to the observed reduction in functional connectivity.

By contrast, the QMT group showed increased synchronization in the precentral and postcentral gyri and in the angular gyrus, a higher-order associative region involved in visuospatial attention and cognitive processing. Of note, the angular gyrus has previously shown significant morphological changes following QMT in healthy subjects [20], lending further support to its relevance as a potential target of training-related plasticity. Importantly, QMT did not simply increase connectivity in selected regions; rather, it was associated with relative stability in networks that showed reduced connectivity in the SHAM group. This broader pattern is consistent with previous studies showing that motor training in PD can induce increases in resting-state connectivity that are functionally meaningful [5,6,7,8,9,10,11,42,76,77]. Taken together, these findings support the interpretation that the present results reflect a differential network response to the two training conditions, rather than short-term neuroprotection in a disease-modifying sense. QMT appears to favor task-relevant network engagement and selective reorganization in systems involved in motor control and higher-order cognitive processing.

The between-group comparison of delta maps further reinforced this interpretation, showing lower functional connectivity in the control group than in the QMT group within specific networks, especially the sensorimotor and cerebellar systems. Within the sensorimotor network, local maxima were identified in the right precentral gyrus and in the SMA. The involvement of the SMA is particularly noteworthy, given its central role in postural control, balance, and the temporal ordering of motor sequences [86]. SMA function is especially relevant for internally generated movements rather than externally triggered actions [87], and altered SMA connectivity is considered a major contributor to the motor deficits observed in PD. In line with this, non-invasive brain stimulation targeting the SMA has been extensively investigated in randomized clinical trials and has shown efficacy in ON-state PD patients [88]. The predominantly right-sided effects observed in the present study are not unexpected in the context of QMT, as prior electrophysiological evidence indicates that QMT can induce hemisphere-specific connectivity changes depending on condition and duration, with distinct intra- and interhemispheric modulations [8].

The cerebellar findings deserve special attention. We observed decreased BOLD synchronization in the control group and relatively increased connectivity in the QMT group in the inter-group analysis. The cerebellum plays a crucial role in movement preparation and execution and is also involved in the precise timing of self-paced actions [89]. In PD, cerebellar recruitment is thought to compensate, at least in part, for basal ganglia dysfunction and to support movement velocity [90]. From this perspective, the present results are compatible with the hypothesis that QMT may facilitate or preserve compensatory cerebellar mechanisms in PD. This point is especially relevant because the cerebellum has long been regarded as a key compensatory node in the disease [91]. Because QMT requires continuous updating of body position in space, response monitoring, and coordinated sequencing of movements without visual cues, it is plausible that cerebellar circuits are particularly engaged by this form of practice. The preservation of cerebellar connectivity may therefore represent one of the neural substrates underlying the potential rehabilitative value of QMT.

In contrast with the functional connectivity results, the exploratory radiomics analysis yielded only weak evidence of measurable pre-post differences. In this part of this study, we assessed whether whole-brain radiomic signatures derived from morphological tissue-informative MR images, namely T1-weighted MPRAGE and FA maps, could detect subtle differences between pre- and post-QMT conditions in PD. Overall, the best-performing model, a support vector machine, achieved only modest discrimination in internal testing, with a ROC-AUC of 0.65, while classification accuracy and F1-score remained close to chance. In addition, none of the selected predictors survived correction for multiple comparisons, suggesting that any detectable pre-post signal was small and likely heterogeneous across individuals. Accordingly, these findings support a cautious interpretation in terms of feasibility rather than efficacy [61,66].

The modest sensitivity of the whole-brain radiomics approach is also biologically plausible. In PD, diffusion MRI studies have reported localized white-matter changes after 4 weeks of gait training rather than widespread structural reorganization [23]. Similarly, a controlled cognitive training study found no effects on whole-brain tract-based metrics or global network topology, but did report subtle local microstructural changes in a tract of interest, namely the anterior thalamic radiation [24]. Prior work on QMT in healthy subjects has also supported the biological plausibility of training-related brain modulation by showing electrophysiological changes, including increased alpha coherence and connectivity, together with diffusion MRI microstructural changes after weeks of practice [8,19,21]. Taken together, these observations suggest that the weak radiomic results observed here may reflect the limited sensitivity of whole-brain, intensity- and texture-based descriptors to capture localized neuroplastic changes. If QMT-related adaptations are focal, network-specific, or tissue-compartment specific, averaging radiomic information across the whole brain may dilute the relevant signal.

This interpretation is further supported by previous evidence indicating that QMT-related structural adaptations are not diffuse, but regionally specific. Prior studies have reported cerebellar volume changes and white-matter effects after QMT, pointing toward anatomically circumscribed rather than global structural modulation. In line with this, a recent landmark-free cortical surface morphometric study described QMT-related shape variations in motor and associative cortical regions [20], supporting the use of surface-based markers when the aim is to quantify training-induced plasticity. Similarly, the discrepancy between the limited FA-based radiomic discrimination observed here and prior longitudinal findings suggests that white-matter effects may be better captured through diffusion-specific tract- or voxel-wise approaches rather than by whole-brain radiomic summarization [19]. Methods such as tract-based spatial statistics, tractography-based analyses, pixel-based analysis, or microstructural models beyond the diffusion tensor may therefore provide greater sensitivity and anatomical specificity for detecting subtle training-related changes, especially in heterogeneous clinical populations such as PD. More broadly, these results indicate that future studies should prioritize anatomically targeted, VOI-based, surface-based, tract-specific, or multimodal imaging strategies rather than relying exclusively on whole-brain radiomic summaries. In particular, VOI-based radiomics focused on the cerebellum and SMA/sensorimotor regions may provide a more sensitive framework for detecting localized training-related effects in PD.

Taken together, the rs-fMRI and radiomics findings suggest an important interpretative point. Over a relatively short intervention interval, QMT-related neuroplasticity in PD may be more readily detectable at the level of large-scale functional interactions than through global structural image descriptors. This is consistent with the possibility that functional reorganization may precede, exceed, or occur independently from structural changes measurable with whole-brain radiomic methods. It also supports the use of rs-fMRI as a particularly sensitive tool for investigating training-related modulation in this context [30]. Indeed, recent work quantifying the mapping between electrocorticography and functional MRI recordings in humans supports the biological plausibility of detecting subtle training-related modulation with MRI-derived measures [37]. In this perspective, different neuroimaging modalities may capture distinct spatial and temporal scales of intervention-induced plasticity, and early functional adaptations should not necessarily be expected to coincide with detectable global radiomic differences.

The present study has several strengths. It should be viewed in the context of a still limited and heterogeneous MRI intervention literature in PD, and, to our knowledge, represents the first prospective MRI investigation of QMT in this population. Most notably, it used a randomized longitudinal design and included a patient group receiving a control intervention, thereby enabling the comparison of two distinct training conditions across the same time window. This strengthens the interpretation of the functional findings by showing that preserved or enhanced connectivity was not simply a generic consequence of repeated measurement or passage of time. In addition, this study explored the feasibility of an IBSI-compliant radiomics workflow on standard clinical acquisitions, thereby providing useful methodological information even in the absence of strong discriminative performance. This aspect is not trivial. Feasibility studies can be informative even when they yield limited discrimination, particularly in fields where biomarker development is vulnerable to false discovery and overinterpretation. Recent reviews on imaging biomarker development have emphasized the importance of rigorous evaluation and transparent reporting, and have explicitly noted that publishing negative or limited findings may be valuable because it discourages unproductive biomarker directions and helps refine future study design and validation strategies [92].

Several limitations should be acknowledged. First, this study was conducted on a clinical 1.5T scanner, which provides a lower signal-to-noise ratio than higher-field systems. Although the longitudinal design and intra-individual comparisons partly mitigate this limitation, reduced image quality may nonetheless have affected sensitivity, particularly for subtle radiomic effects. Second, while rs-fMRI captured measurable changes in functional connectivity, these neural changes were not paralleled by statistically significant short-term changes in the available clinical measures over the 4-week observation period. This was not unexpected given the brevity of the intervention and the limited sensitivity of conventional clinical scales to subtle early neuroplastic effects. Accordingly, the present findings should be interpreted as evidence of early network-level modulation that was not yet clinically appreciable, rather than as proof of immediate clinical efficacy. Longer studies integrating prespecified clinical, motor, cognitive, and imaging endpoints will be required to determine whether the observed neural effects become clinically meaningful over time. Third, the radiomics sample size was relatively small, and no non-intervention control group was available for that analysis, limiting the ability to disentangle training-related effects from test-retest variability, disease progression, or scanner-related fluctuations. More broadly, although the randomized longitudinal design was appropriate for the rs-fMRI objective, the sample size and single-center enrollment limit the representativeness of the cohort and are insufficient for definitive radiomics-based biomarker development or robust machine-learning generalization. Fourth, the pre-post radiomics design introduced within-subject dependence, whereas the modelling strategy was implemented at the scan level; therefore, the reported performance should be interpreted as an exploratory within-cohort estimate rather than as evidence of subject-level generalizability. The same caution applies to the univariate radiomics analyses, which should be regarded as descriptive rather than as paired subject-level inference. Fifth, the use of whole-brain volumes of interest may have diluted focal or network-specific alterations by averaging across large and heterogeneous tissue compartments. Accordingly, the radiomics results should be interpreted strictly as exploratory feasibility findings. Future studies should prioritize anatomically targeted VOI-based radiomics strategies, particularly in regions that emerged as functionally relevant in the present study, such as the cerebellum and SMA/sensorimotor areas. They should also adopt subject-level longitudinal designs based on post-pre delta features, paired multivariate modelling, or mixed-effects approaches that explicitly account for repeated measurements within the same individual. Additional limitations include the absence of an active cognitive control condition, which would have helped isolate the specific contributions of motor and cognitive engagement in QMT, and the possibility that individual differences in disease progression and neurodegeneration influenced responsiveness to training. Also, this study was not designed or powered to evaluate QMT effects according to PD clinical subtypes; future studies should prospectively examine whether subtype-specific phenotypes show differential neural or clinical responsiveness to this intervention.

Despite these limitations, the present findings support the relevance of QMT as a potentially scalable and accessible intervention in PD. QMT is designed to enhance attention, coordination, and mindful action through the integration of motor response and cognitive processing, requiring participants to navigate verbal instructions within a defined space without visual cues. By encouraging the suspension of automatic habits, the direction of attention, and greater receptivity to sensorimotor demands, QMT may promote stability and adaptability in neural systems that extend beyond those directly involved in the overt motor task. Importantly, it can be practiced at home in short sessions and with a relatively rapid learning curve, which makes it particularly attractive from a rehabilitation perspective.

## 5. Conclusions

In conclusion, the present study provides preliminary evidence that QMT may support relative functional stability and selective reorganization of resting-state functional connectivity in PD, particularly within sensorimotor, frontoparietal, and cerebellar networks, while whole-brain radiomic features derived from T1-weighted and FA images appear to have limited sensitivity for detecting short-term training-related changes in this setting. These findings suggest that functional network measures may currently offer greater sensitivity than global structural radiomics for capturing early QMT-related neuroplastic effects in PD. Overall, this study may help inform future investigations of motor-cognitive training in PD and contribute to refining the imaging strategies most suitable for tracking its effects.

## Figures and Tables

**Table 1:** Demographic, clinical, and significant psychological features of PD patients included in the control and QMT groups. Data are presented as median (min–max range) or mean (standard deviation).

	Control Group (*n* = 25)	QMT Group (*n* = 23)	*p* < 0.05
**Demographics**			
Age (years)	65 (47–75)	69 (46–82)	n.s.
Sex (M/F)	15/10	12/13	n.s.
Handedness (Right/Left)	23/2	22/1	n.s.
Self-efficacy Questionnaire	52.9 (8.7)	50.0 (10.0)	n.s.
**Neuropsychological Test**			
MoCA adjusted score	24.7 (3.1)	23 (4.0)	n.s.
MoCA equivalent score	4 (0–4)	4 (0–4)	n.s.
MCI (yes/no)	8/17	11/12	n.s.
**Clinical Features**			
Hoehn & Yahr scale	2 (1–2.5)	2 (1–3)	n.s.
Disease Duration (years)	5 (1–10)	4 (2–12)	n.s.
LEDD	398 (193)	454 (276)	n.s.
UPDRS I	4 (0–11)	4.5 (1–19)	n.s.
UPDRS II	3 (0–11)	4.5 (1–22)	n.s.
UPDRS III	15 (3–39)	19.5 (4–29)	n.s.
UPDRS IV	0 (0–5)	0 (0–10)	n.s.
Time Up & Go Test (sec)	9.1 (1.4)	9.7 (2.1)	n.s.
Patient with Falls 1 month prior	0/25	2/23	n.s.

n.s. = not significant.

**Table 2:** RSNs showing significant FC reduction after 4 weeks of SHAM training in PD patients (*p* < 0.01, FDR corrected). Peak MNI coordinates (mm) within clusters were identified using the minimum peak distance between the local maxima of 20 mm. Anatomical localizations of peak MNI coordinates were established according to the Harvard–Oxford Cortical Structural Atlas.

		MNI Coordinates			
Cluster Size (Voxels)	T	x	y	z	Cluster Location (Local Maxima)
Default mode network					
210	5.03	−8	56	38	L Frontal Pole
	3.50	2	64	20	R Frontal Pole
115	4.55	50	8	−36	R Temporal Pole
Dorsal attention network					
332	5.03	26	−66	56	R Lateral Occipital Cortex, superior division
	3.18	38	−46	64	R Superior Parietal Lobule
167	4.57	−30	−62	60	L Lateral Occipital Cortex, superior division
139	4.99	48	2	32	R Precentral Gyrus
124	5.29	−26	0	62	L Superior Frontal Gyrus
Executive control network					
187	3.72	0	26	32	Paracingulate Gyrus
Occipital network					
144	4.45	38	−68	−16	R Occipital Fusiform Gyrus
Sensorimotor network I					
846	4.50	−32	−34	66	L Postcentral Gyrus
	4.39	−14	−56	66	L Superior Parietal Lobule
	4.30	−2	−32	74	L Precentral Gyrus
361	4.80	8	−60	64	R Precuneous Cortex
	4.35	32	−42	66	R Superior Parietal Lobule
267	4.52	−2	−10	58	L Juxtapositional Lobule Cortex (formerly Supplementary Motor Cortex)
188	4.61	−30	−8	50	L Precentral gyrus
Sensorimotor network II					
316	4.38	66	−14	36	L Postcentral Gyrus
Cerebellar network					
166	4.89	−20	−86	−46	L Cerebellar Crus II
	2.87	−30	−78	−26	L Cerebellar Crus I
119	3.87	0	−64	−20	Vermis Lobule VI
	3.44	−10	−44	−34	Brainstem

**Table 3:** RSNs showing significant FC increase after 4 weeks of QMT in PD patients (*p* < 0.01, FDR corrected). Refer to Table 2 for a detailed explanation of the table layout.

		MNI Coordinates			
Cluster Size (Voxels)	T	x	y	z	Cluster Location (Local Maxima)
Right Frontoparietal network					
101	3.99	60	−50	38	R Angular Gyrus
Sensorimotor network II					
104	3.62	64	−12	32	R Postcentral Gyrus
	3.01	56	6	36	R Precentral Gyrus

**Table 4:** Significant FC differences between ∆FC maps of SHAM and QMT groups of PD patients (*p* < 0.01, FDR corrected). ∆FC in SHAM patients was significantly lower than ∆FC in QMT patients. Refer to Table 2 for a detailed explanation of the table layout.

		MNI Coordinates			
Cluster Size (Voxels)	T	x	y	z	Cluster Location (Local Maxima)
Sensorimotor network I					
115	4.41	−20	−42	64	L Postcentral Gyrus
100	3.72	2	−4	68	R Juxtapositional Lobule Cortex (formerly Supplementary Motor Cortex)
Cerebellar network					
102	3.83	−18	−86	−34	L Cerebellar Crus II

**Table 5:** Model of three ensembles of Random Forest classifiers. Classification performance in terms of ROC-AUC, Accuracy, Sensitivity, Specificity, PPV, NPV, F1 score, corresponding 95% confidence interval, and statistical significance with respect to chance/random classification (*p*-value). Performance is reported for training, validation, and internal testing sets.

	Training (50% Threshold)	Validation (50% Threshold)	Internal Testing (50% Threshold)	Internal Testing (46% Threshold)
ROC-AUC (%) [95%]	100 ** [100–100]	60 ** [56–64]	62 * [50–74]	54
Accuracy (%) [95%]	100 ** [100–100]	53 ** [51–56]	49 [42–57]	48
Sensitivity (%) [95%]	100 ** [100–100]	51 [46–55]	45 [31–59]	50
Specificity (%) [95%]	100 ** [100–100]	56 ** [52–61]	53 [38–69]	45
PPV (%) [95%]	100 ** [100–100]	55 * [51–59]	50 [36–64]	48
NPV (%) [95%]	100 ** [100–100]	54 * [51–58]	46 [34–58]	47
F1 score (%) [95%]	100 ** [100–100]	46 [42–49]	40 [28–52]	49

* *p*-value < 0.05/** *p*-value < 0.005.

**Table 6:** Model of three ensembles of Support Vector Machine classifiers. Classification performance in terms of ROC-AUC, Accuracy, Sensitivity, Specificity, PPV, NPV, F1 score, corresponding 95% confidence interval, and statistical significance with respect to chance/random classification (*p*-value). Performance is reported for training, validation, and internal testing sets.

	Training (50% Threshold)	Validation (50% Threshold)	Internal Testing (50% Threshold)	Internal Testing (51% Threshold)
ROC-AUC (%) [95%]	72 ** [72–73]	66 ** [62–69]	65 * [54–76]	57
Accuracy (%) [95%]	66 ** [65–66]	56 ** [53–58]	49 [42–56]	48
Sensitivity (%) [95%]	65 ** [64–65]	54 * [50–59]	52 [38–65]	50
Specificity (%) [95%]	67 ** [66–68]	57 ** [53–61]	47 [32–61]	45
PPV (%) [95%]	66 ** [66–67]	58 ** [54–61]	50 [39–61]	48
NPV (%) [95%]	65 ** [65–66]	57 ** [53–61]	46 [34–58]	47
F1 score (%) [95%]	65 ** [65–66]	48 [45–52]	45 [34–55]	49

* *p*-value < 0.05/** *p*-value < 0.005.

**Table 7:** Model of three ensembles of K-Nearest Neighbors classifiers. Classification performance in terms of ROC-AUC, Accuracy, Sensitivity, Specificity, PPV, NPV, F1 score, corresponding 95% confidence interval, and statistical significance with respect to chance/random classification (*p*-value). Performance is reported for training, validation, and internal testing sets.

	Training (50% Threshold)	Validation (50% Threshold)	Internal Testing (50% Threshold)	Internal Testing (48% Threshold)
ROC-AUC (%) [95%]	61 ** [60–62]	62 ** [59–65]	64 * [54–75]	54
Accuracy (%) [95%]	58 ** [57–59]	55 ** [52–58]	53 [46–59]	55
Sensitivity (%) [95%]	57 ** [56–59]	56 * [51–60]	35 [21–49]	50
Specificity (%) [95%]	59 ** [57–60]	55 * [51–59]	70 ** [58–82]	60
PPV (%) [95%]	59 ** [58–60]	58 ** [54–62]	52 [35–68]	56
NPV (%) [95%]	58 ** [57–59]	57 ** [53–61]	55 [48–62]	55
F1 score (%) [95%]	57 ** [56–58]	50 [47–54]	34 [21–46]	53

* *p*-value < 0.05/** *p*-value < 0.005.

**Table 8:** Model of three ensembles of Multi-Layer Perceptron classifiers. Classification performance in terms of ROC-AUC, Accuracy, Sensitivity, Specificity, PPV, NPV, F1 score, corresponding 95% confidence interval, and statistical significance with respect to chance/random classification (*p*-value). Performance is reported for training, validation, and internal testing sets.

	Training (50% Threshold)	Validation (50% Threshold)	Internal Testing (50% Threshold)	Internal Testing (49% Threshold)
ROC-AUC (%) [95%]	80 ** [78–83]	71 ** [68–75]	57 [48–67]	53
Accuracy (%) [95%]	76 ** [74–78]	61 ** [58–63]	49 [42–57]	45
Sensitivity (%) [95%]	76 ** [74–78]	61 ** [57–65]	48 [35–62]	45
Specificity (%) [95%]	75 ** [73–78]	60 ** [56–65]	50 [35–65]	45
PPV (%) [95%]	77 ** [74–79]	64 ** [60–67]	49 [38–61]	45
NPV (%) [95%]	75 ** [73–78]	65 ** [61–69]	46 [34–57]	45
F1 score (%) [95%]	76 ** [74–78]	55 * [51–58]	43 [31–54]	45

* *p*-value < 0.05/** *p*-value < 0.005.

**Table 9:** Model of three ensembles of Logistic Regression classifiers. Classification performance in terms of ROC-AUC, Accuracy, Sensitivity, Specificity, PPV, NPV, F1 score, corresponding 95% confidence interval, and statistical significance with respect to chance/random classification (*p*-value). Performance is reported for training, validation, and internal testing sets.

	Training (50% Threshold)	Validation (50% Threshold)	Internal Testing (50% Threshold)	Internal Testing (53% Threshold)
ROC-AUC (%) [95%]	73 ** [73–74]	66 ** [62–69]	65 * [54–76]	55
Accuracy (%) [95%]	67 ** [66–67]	54 ** [52–57]	50 [44–56]	53
Sensitivity (%) [95%]	67 ** [66–67]	56 * [51–60]	53 [40–67]	50
Specificity (%) [95%]	67 ** [66–68]	53 [49–57]	47 [32–61]	55
PPV (%) [95%]	67 ** [66–68]	55 ** [52–59]	51 [40–63]	53
NPV (%) [95%]	67 ** [66–67]	56 ** [52–60]	49 [36–61]	52
F1 score (%) [95%]	67 ** [66–67]	49 [45–52]	46 [35–56]	51

* *p*-value < 0.05/** *p*-value < 0.005.

**Table 10:** Ensemble of Support Vector Machine classifiers. The 6 selected predictors, sorted in descending order according to their statistical significance.

	Feature Family	Feature Nomenclature	Median in the Negative Class [95% CI]	Median in the Positive Class [95% CI]	Uncorrected *p*-Value	Corrected *p*-Value
I	Neighbourhood Grey Tone Difference Matrix	T1-weighted—Logarithm Strength	4.57 × 10^−2^ [4.27 × 10^−2^–4.87 × 10^−2^]	4.22 × 10^−2^ [3.84 × 10^−2^–4.60 × 10^−2^]	0.10	0.61
II	Intensity-Based Statistics	FA—Logarithm Variance	1.33 × 10^3^ [1.25 × 10^3^–1.41 × 10^3^]	1.22 × 10^3^ [1.08 × 10^3^–1.37 × 10^3^]	0.25	1.00
III	Neighbourhood Grey Tone Difference Matrix	FA—Busyness	4.33 × 10^1^ [4.12 × 10^1^–4.54 × 10^1^]	4.30 × 10^1^ [4.15 × 10^1^–4.46 × 10^1^]	0.56	1.00
IV	Grey-Level Co-Occurrence Matrix	T1-weighted—Squareroot Sum Variance	6.32 × 10^2^ [5.96 × 10^2^–6.68 × 10^2^]	5.95 × 10^2^ [5.49 × 10^2^–6.40 × 10^2^]	0.74	1.00
V	Intensity Histogram	FA—Logarithm Uniformity	2.77 × 10^−1^ [2.49 × 10^−1^–3.05 × 10^−1^]	3.02 × 10^−1^ [2.79 × 10^−1^–3.24 × 10^−1^]	0.76	1.00
VI	Neighbouring Grey Level Dependence Matrix	FA—Logarithm High Dependence Emphasis	3.26 × 10^2^ [3.05 × 10^2^–3.46 × 10^2^]	3.37 × 10^2^ [3.21 × 10^2^–3.53 × 10^2^]	0.82	1.00

## Data Availability

The data presented in this study are available on request from the corresponding author due to privacy reasons.

## Abbreviations

The following abbreviations are used in this manuscript:

AUC	Area Under the Curve
BOLD	Blood-oxygen-level-dependent
BS	Brier Score
CI	Confidence Interval
CSF	Cerebrospinal fluid
ECE	Expected Calibration Error
EPI	Echo-planar imaging
FA	Fractional Anisotropy
FC	Functional connectivity
FSL	FMRIB Software Library
FDR	Fisher Discriminant Ratio
GLCM	Gray-Level Co-occurrence Matrix
GLRLM	Gray-Level Run Length Matrix
GLSZM	Gray-Level Size Zone Matrix
GM	Gray matter
GRE	Gradient echo
ICA	Independent component analysis
IBSI	Image Biomarker Standardisation Initiative
LEDD	Levodopa equivalent daily dose
MDS-UPDRS	Movement Disorder Society-sponsored Unified Parkinson’s Disease Rating Scale
MNI	Montreal Neurological Institute
MoCA	Montreal Cognitive Assessment
MPRAGE	Magnetization-Prepared Rapid Gradient-Echo
MRI	Magnetic Resonance Imaging
NGLDM	Neighboring Gray Level Dependence Matrix
NGTDM	Neighborhood Gray Tone Difference Matrix
NPV	Negative Predictive Value
PCA	Principal Component Analysis
PD	Parkinson’s Disease
PPV	Positive Predictive Value
QMT	Quadrato Motor Training
rs-fMRI	Resting-state functional magnetic resonance imaging
RSN	Resting-state network
ROC	Receiver Operating Characteristic
SMA	Supplementary motor area
SPSS	Statistical Package for the Social Sciences
VOI	Volume of Interest
WM	White matter

## Appendix A

Whole-brain rs-fMRI was acquired with the following parameters: Repetition Time = 2500 msec, Echo Time = 59 msec, Field Of View = 28 × 28 cm, flip angle = 84°, voxel size = 2.2 × 2.2 × 2.2 mm, acceleration iPAT = 4. Field mapping was obtained using a double echo GRE T2* weighted sequence (Repetition Time = 586 msec, Echo Time1/Echo Time2 = 4.76/9.52 msec, Field Of View = 19 × 19 cm, flip angle = 60°, voxel size = 3 × 3 × 3 mm).

The anatomical imaging protocol included a 3D T1-weighted MPRAGE acquisition with the following parameters: Repetition Time = 1750 ms, Echo Time = 3.15 ms, Inversion Time = 1000 ms, flip angle = 8°, acquisition matrix = 256 × 256, number of excitations = 2, Field Of View = 25 × 25 cm, isotropic voxel size = 1 × 1 × 1 mm, and parallel imaging acceleration factor (phase-encoding) = 2.

Diffusion-weighted imaging data were acquired to derive FA maps using the following parameters: TR = 7100 ms, TE = 63 ms, Field Of View = 25 × 25 cm, isotropic voxel size = 2 × 2 × 2 mm, b-value = 1000 s/mm^2^, 30 diffusion-encoding directions, and one b0 (non-diffusion-weighted) volume.
